# Catalytic Performance of Sn-β Zeolites with Different Si/Al Ratios in the Conversion of Glucose to Lactic Acid

**DOI:** 10.3390/molecules29235707

**Published:** 2024-12-03

**Authors:** Xiaowei Zhuang, Yongshun Feng, Hui Qiao, Weiming Yang, Xin Pan

**Affiliations:** Zhejiang Academy of Forestry, Liuhe Road 399, Hangzhou 310023, China; zhuangxiaowei@zjforestry.ac.cn (X.Z.); fengyongshun@zjforestry.ac.cn (Y.F.); qiaohui@zjforestry.ac.cn (H.Q.); yangweiming@zjforestry.ac.cn (W.Y.)

**Keywords:** Sn-β zeolite, lactic acid, acid catalysis, Si/Al ratio, glucose conversion

## Abstract

Lactic acid is an important platform feedstock for synthesizing various chemicals. Lactic acid is normally converted from any sugar such as glucose, and Sn-β zeolite is an effective catalyst. In this study, β zeolite with different Si/Al ratios was prepared and characterized. Sn precursor is reacted with β zeolite by high-energy mixing and introduced into the framework of β zeolite to obtain Sn-β zeolite with different Si/Al ratios. The physicochemical properties of Sn-β zeolite were characterized by XRD, FTIR, N_2_ physical adsorption, UV Vis diffuse reflectance spectroscopy, and pyridine adsorption FTIR. The results showed that when the Si/Al molar ratio of β zeolite was less than 45, the skeleton load of Sn in β zeolite increased effectively with the decrease in aluminum content, and the Lewis acid and Brønsted acid site numbers could be improved. As the Si/Al ratio exceeded 45, the increase in Sn load in β zeolite slowed down, and the Lewis acid and Brønsted acid site numbers were decreased. The results from the catalytic conversion of glucose to lactic acid confirmed that the too high Si/Al ratio caused a decrease conversion rate. The highest performance of the prepared Sn-β zeolites with the highest catalytic efficiency had a glucose conversion rate of 96.69% and lactic acid yield of 39.42% within 7 h at 190 °C in a pressure reactor.

## 1. Introduction

Facing the global carbon emissions caused by fossil energy consumption, the transformation of renewable biomass into value-added chemicals has attracted much attention. Among the chemical substances obtained from biomass resources, lactic acid as an important platform molecule compound has a wide range of applications in the chemical, pharmaceutical, and food industries [1]. Currently, the market demand for lactic acid also shows a large-scale growth. It is estimated that the global demand for lactic acid will reach 1.96 million tons in 2025 [2]. The existing production process of lactic acid primarily involves sugar fermentation. Due to the severe pollution and low utilization rate of raw materials, it is necessary to develop a new environmentally friendly lactic acid production process.

In 1967, Mobil Corporation of the United States first developed a large-pore high-silica zeolite with a three-dimensional twelve-membered channel structure, which was named β zeolite [3]. Due to its special pore structure, β zeolite has excellent performance in catalytic reactions [4], and is widely used in petroleum refining [5] and petrochemical processes such as hydrocracking, isomerization, alkylation, and dewaxing [6]. Many researchers have found that using Sn atoms to replace silicon or aluminum atoms on the β zeolite framework can construct Lewis acidic sites in β zeolite, which exhibit unique catalytic properties [7]. The Lewis acid in Sn-β zeolite has been proven to have good catalytic performance in the Baeyer–Villiger reaction [8] and Meerwein–Ponndorf–Verley reaction [9]. It shows high activity and selectivity in the isomerization of trisaccharide to lactic acid and the formation of lactic acid esters through isomerization–esterification [10].

However, under normal conditions, it is difficult for Sn atoms to replace silicon or aluminum atoms in the framework of β zeolite. It is necessary to remove the central atoms in the framework of β zeolite first to vacate the space of the β zeolite framework for Sn to enter [11]. Therefore, the synthesis of β zeolite with a high Si/Al ratio has been the focus of research in recent years. The traditional method of synthesizing zeolite containing Al in an alkaline medium using TEA as a template was not successful in preparing pure Si zeolite structure, because the nucleation step of crystallization requires aluminum species [12]. Corma et al. developed an aluminum-free β zeolite synthesis method, which has been proven to be very effective in increasing the Sn loading in zeolite. However, this method required the use of HF, which was harmful to human health. In addition, the crystallization time of the zeolite was 20 days, which seriously hindered its commercial application [13]. Yang et al. further improved the method based on Corma’s research so that the crystallization time of zeolite was shortened to about 5 days, and the seed-assisted synthesis of Sn was needed in the synthesis process, while the synthesis of Si β seed took one day [14]. Furthermore, Yang et al. shortened the crystallization time of zeolite to less than 3 days through zeolite nano crystallization technology, but the processing process also became complex [15], and the existing silica zeolite methods almost all need to use HF [16], so the whole synthesis process is still not suitable for commercial use.

In addition, because the first step in the catalytic conversion of glucose to lactic acid is isomerization, it is necessary to add a certain amount of alkali or directly change the reaction system into an alcohol phase to achieve the effect of efficient catalysis [17]. Either adding alkali or changing the reaction system made the whole catalytic process environmentally unfriendly [18]. Therefore, based on the Sn-β zeolite catalyst, surface modification was an effective way to improve the performance of the catalyst [19,20]. Meanwhile, Al has also been confirmed to have the ability to catalyze the hydrothermal conversion of glucose into lactic acid [19,21,22].

Compared with synthetic molecular sieves, natural zeolite has the advantages of abundant sources, low cost, and physical properties that are not weaker than synthetic molecular sieves, but they have the disadvantages of high Al content in zeolite framework and difficult introduction of heteroatoms. In this study, natural β zeolite was used as raw material to control the aluminum content in β zeolite through different pickling processes. Through physical high-energy grinding, Sn was fully mixed with β zeolite and forced closer to the framework of β zeolite by mechanical extrusion. This makes it easier to introduce Sn into the framework of β zeolite by subsequent high-temperature calcination, and Sn-β zeolite catalysts with different Si/Al ratios were prepared. The catalytic performance of the catalyst for the conversion of glucose to lactic acid was investigated by hydrothermal method. Through this study, it is expected to obtain a low-cost high-performance catalyst with a Sn-β-Al structure.

## 2. Results and Discussion

### 2.1. Structure Analysis of the Sn-β Zeolites

The mass percentages of Si, Al, and Sn in the dealuminated β zeolites are shown in Table 1. As the concentration of the nitric acid increased from 0.2 mol/L to 1.0 mol/L, the aluminum content decreased from 2.56% to 0.42%, leading to an increase in the Si/Al ratio (molar ratio, the same as below). The acid washing could effectively remove the aluminum in the β zeolites whereas Sn tended to integrate into the β zeolite framework more readily. Consequently, Sn-β zeolite with a high Sn/Al ratio can effectively enhance the loading capacity of catalyst elements. Notably, when the Si/Al ratio was below 32, an increase in the Si/Al ratio significantly enhanced the loading capacity for Sn.

The loading capacity of the SA3 sample was 2.5 and 5.4 times higher than that of SA2 and SA1, respectively. Meanwhile, the Sn loading of the SA5 sample was 1.03 times that of SA4, indicating that when the Si/Al ratio surpassed 45, further acid washing for dealumination could not enhance the loading capacity for Sn. It indicated that once the removal of aluminum from the β zeolite framework reached a certain extent, the Sn element could not fully occupy the vacancies due to the mismatch in atomic radius between Sn and Al. Therefore, it was economically unwise to excessively remove aluminum in pursuit of a high loading rate of target elements in Sn-β zeolite.

**Figure 1:**
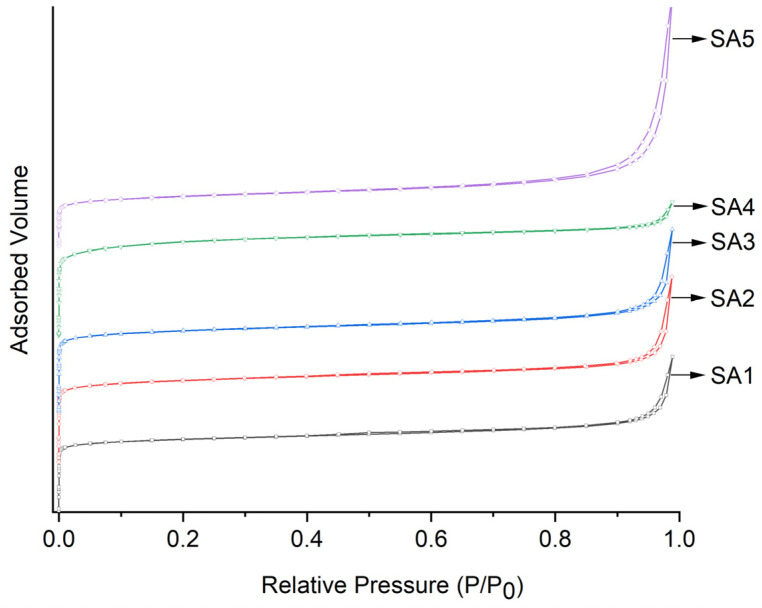
N_2_ physical adsorption–desorption isotherm curves of different Sn-β zeolites.

The nitrogen physisorption isotherms are shown in Figure 1. It can be found that all the samples exhibited characteristics of type I adsorption isotherms despite the different Si/Al ratios. These samples demonstrated high adsorption capacities in the low specific pressure region, indicating the presence of a well-developed microporous structure. The appearance of a hysteresis loop at P/P0 > 0.70 suggested the presence of a certain amount of mesopores in the Sn-β zeolite samples. Table 2 lists the specific surface areas (S_BET_) of various catalysts. The specific surface area and pore volume (V_p total_) generally increased after the dealumination and Sn loading of the zeolites. Similarly, the specific surface area decreased whereas the pore volume continued to increase with excessive dealumination. This might be the reason for the formation of many interparticle pores due to the disorderly accumulation of small-grain Sn-β zeolites after excessive dealumination.

**Figure 2:**
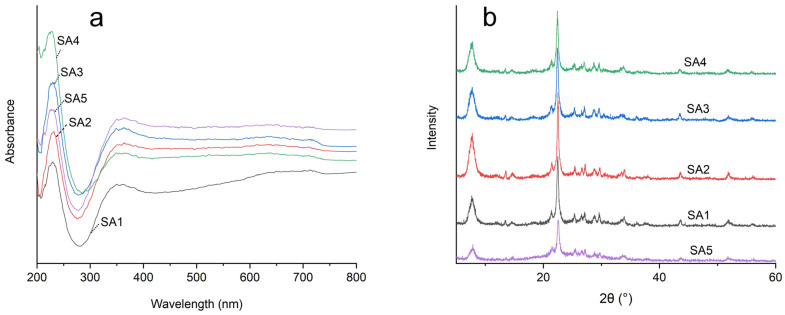
(**a**) UV spectra of different Sn-β zeolites and (**b**) XRD patterns of different Sn-β zeolites.

UV diffuse reflectance spectroscopy primarily serves to ascertain the charge transfer and d-d transitions of metal ions on the catalyst surface. In complexes formed by transition metal ions and ligands, the absorption spectrum arises when electrons are transferred from the electron donor to the electron acceptor, constituting a charge transfer absorption spectrum. Since this charge transfer requires high energy, the absorption band predominantly emerges in the ultraviolet region. If transition metal ions undergo d-orbital transitions due to ligand influence, the absorption band emerges in the visible region, occasionally even in the infrared region owing to the low energy required for d-electron transitions [23]. By analyzing these two types of spectrums, the valence state and coordination symmetry of metal ions could be determined. The UV spectra of different Sn-β zeolites with different Si/Al ratios are shown in Figure 2a. It can be found that the Sn-β zeolite samples with different Si/Al ratios all showed strong absorption peaks within the 190–230 nm range; this was similar to Vega-Vila’s research [23]. These peaks resulted from charge transfer when the 2p electron of the bonded oxygen transitioned to the 3d vacancy orbit of the framework heteroatoms, indicating the presence of four coordinating framework heteroatoms. Among these, SA4 demonstrated the strongest absorption peak, followed by SA3, SA2, SA5, and SA1. This suggested that as the degree of aluminum removal from the β zeolite framework increased, the Sn elements tended to enter the central atomic sites of the four-coordinated framework of the β zeolite more easily. However, the excessive removal of aluminum from the central sites of β zeolite might destabilize the four-coordinated framework of the vacancies, leading to structural damage. Consequently, the absorption peak of the SA5 sample at 230 nm was weaker than that of the SA4 sample. The pronounced and broad absorption band spanning from 300 to 480 nm was attributed to the electronic transition signals of non-framework heteroatoms. So, comparing the absorption bands between 300 and 480 nm, it speculated that a too-high loading of the non-framework Sn element loaded in β zeolite could reduce the efficiency of the catalyst.

The monolayer structure of β zeolite was based on the structural unit of the upper layer, translating in different directions to form new multilayer structures. The feature of this structure was that the translation of multi-layer structures did not cause pore blockage, thereby affecting the pore structure of the zeolite. The XRD spectra of Sn-beta zeolites with different Si/Al ratios are shown in Figure 2b. It can be found that the distinct characteristic peaks of β zeolite appeared at 7.6° and 22.4° [24], indicating that the framework structure of β zeolite remained intact after nitric acid treatment. However, the peaks of the SA5 sample were significantly reduced, indicating that excessive dealumination treatment caused a decrease in the crystallinity of Sn-β zeolite. The diffraction peak at 2θ = 26.7° was attributed to the presence of small SnO_2_ particles.

**Figure 3:**
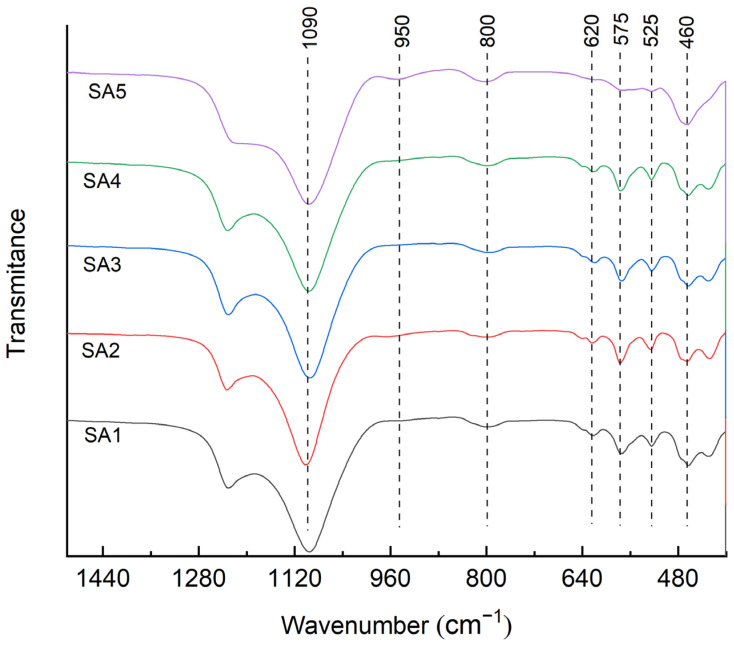
FTIR spectra of different Sn-β zeolites.

The FTIR spectrum of β zeolite is an effective method to analyze whether heteroatoms exist in the framework and the results are shown in Figure 3. The absorption peak at 525 cm^−1^ was attributed to the vibration of double tetrahedral rings in the zeolite framework while the absorption peak at 575 cm^−1^ was attributed to the vibration of pentahedral rings. It could be found that the absorption peak at 525 cm^−1^ of β zeolite in the SA5 sample was smooth and tended to disappear among all the samples. This implied that excessive dealumination could destroy the structure of β zeolite.

The spectral band at approximately 460 cm^−1^ was caused by the bending vibration of the T-O (T is the central atom of zeolites, which can be Si, Al, or Sn) bond; at approximately 620 cm^−1^, it was attributed to the double ring, and at 800 cm^−1^, it was attributed to the symmetric stretching vibration band of TO4. The band at around 1090 cm^−1^ was attributed to the asymmetric stretching mode of Si-O-T. These spectral bands were generally similar across different samples, with only slight shifts. A comparison of the FTIR spectra of SA5 and the other samples showed an increase in the relative intensity of the band at 950 cm^−1^ after excessive dealumination treatment. This band was assigned to the Si-O stretching vibrations of the Si-OH groups present at connectivity defects. Additionally, the decrease in the relative intensity of the band at 525 cm^−1^ in the FTIR spectra of SA5 indicated that the dealumination of the SA5 sample had disrupted the β zeolite structure.

### 2.2. Analysis of Acid and Base Properties of the Sn-β Zeolites

Pyridine-probed FTIR (Py-IR) analysis is a useful tool to identify and quantify the Brønsted and Lewis acid sites in solid acid catalysts. The absorption peak at 1540 cm^−1^ and 1633 cm^−1^ were attributed to the characteristic absorption peak of pyridine complex ions formed by the interaction of pyridine ions with protonic acids, which were used to characterize the Brønsted acid centers of β zeolite. The absorption peaks at 1611 cm^−1^ and 1452 cm^−1^ were attributed to the characteristic absorption peaks of coordination complexes formed by the interaction of pyridine with Lewis acids, which were used to characterize the Lewis acid centers in β zeolite [25]. The peak at 1490 cm^−1^ was a common absorption peak resulting from the interaction of Brønsted and Lewis acids with pyridine ions [26]. Additionally, the absorption peaks at 1596 cm^−1^ and 1445 cm^−1^ were attributed to the characteristic absorption peaks of pyridine ions bound by hydrogen bonds. The absorption peaks at 1581 cm^−1^ were attributed to the characteristic absorption peaks of physically adsorbed pyridine ions.

**Figure 4:**
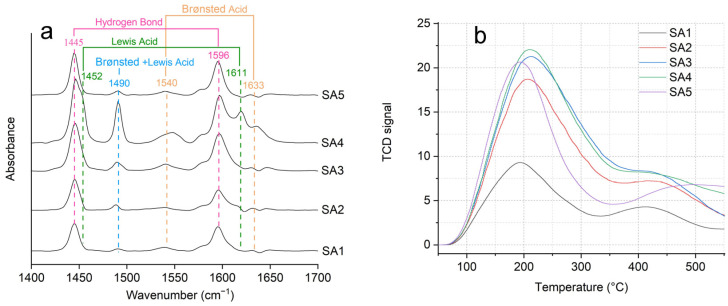
(**a**) Py-IR spectra following the adsorption of pyridine on different Sn-β zeolites and (**b**) NH_3_-TPD curves of different Sn-β zeolites.

It can be seen from Figure 4a and Table 3 that as the Si/Al ratio in the β zeolite increased, the Lewis acids and Brønsted acidity of different catalysts showed a trend of initial increase followed by a decrease. Specifically, when the Si/Al ratio was 45, the Lewis acid and Brønsted acid of the sample reached their peak values. Subsequently, as the Si/Al ratio continued to increase, the Lewis acid and Brønsted acid of the sample showed a downward trend. It indicated that within a certain range, the dealumination treatment of the β zeolite could indeed increase the central vacancies in the β zeolite framework, making it easier for Sn to enter and thus increasing the Lewis acid. The research findings align with those of Shen et al. [27]. Compared to the traditional synthesis method without aluminum, our method was easy to implement and largely increased the number of Lewis acid sites in the β zeolite framework. The increase in Brønsted acid might be attributed to the hydroxyl defects such as Sn-OH and Si-OH caused by acid washing and dealumination. With the further removal of aluminum from the β zeolite, an increase in hydroxyl defects such as Si-OH occurred which led to a decrease in Sn loading, causing both Lewis acid and Brønsted acid to start declining.

The acid strength of the catalysts was determined based on the variation in the adsorption strength over different temperature regions, as analyzed by NH_3_-TPD shown in Figure 4b. The weak acidic sites corresponded to ammonia desorption temperatures below 200 °C, the medium–strong acidic sites at 200–370 °C, and the strong acidic sites at 370–550 °C [28]. With the increase in the Si/Al ratio of the β zeolite and the introduction of Sn, the desorption peak at low and moderate temperatures initially shifted towards the high-temperature region, accompanied by an increase in the peak area. It further shifted towards the low-temperature region, with a decrease in the peak area increase. The quantitative analysis showed that the quantity of weak and medium Lewis acidity determined by the variation in the low- and moderate-temperature desorption peaks could be obtained in the following order: SA4 > SA3 > SA2 > SA5 > SA1 (Table 3). In addition, similar high-temperature desorption peaks were observed for different Sn-β zeolite catalysts, indicating that almost the same trend of strong Lewis acidity was created in the different catalysts. It was interesting to find that the high-temperature desorption peaks of SA5 samples shifted toward higher-temperature regions. These results indicated that a reasonable dealumination and incorporation of Sn into the β-zeolite considerably altered the distribution of acidity, not only increasing the amount of moderate and weak Lewis acidity but also enhancing the amount of strong Lewis acidity. Overall, the surface acidic sites of the variety Sn-β zeolite catalysts were primarily moderate–weak acid sites with a small number of strong acid sites.

### 2.3. Catalytic Conversion of Glucose in the Liquid Phase over Metal-Modified Zeolite Catalysts

**Figure 5:**
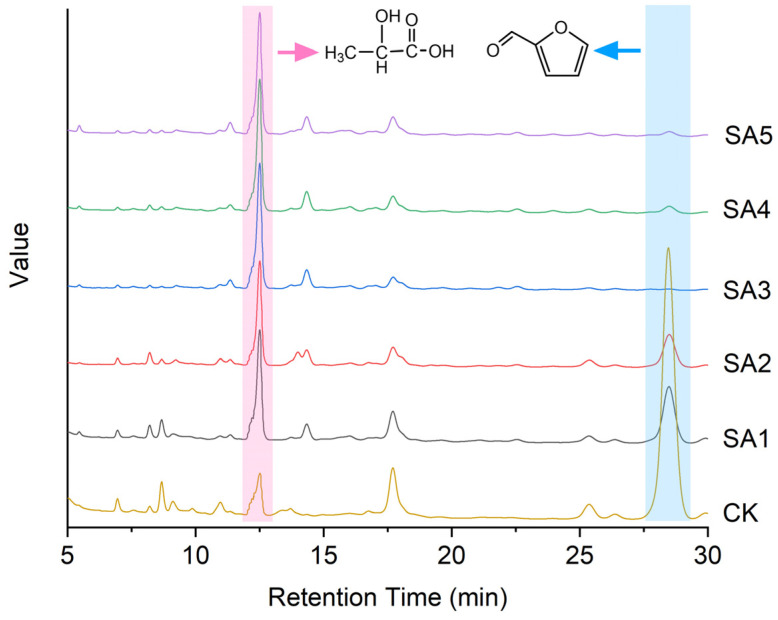
HPLC chromatogram of samples obtained after hydrothermal reaction of glucose with different catalysts.

**Figure 6:**
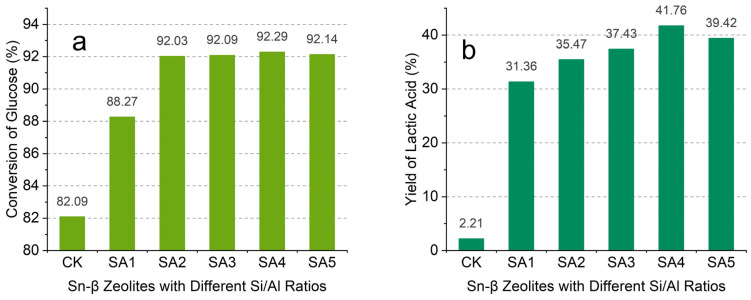
The effect of different catalysts on glucose conversion rate (**a**) and yield of lactic acid (**b**).

Figure 5 shows the HPLC chromatogram of the catalytic products of different catalysts. And, Figure 6 shows the conversion of glucose and the yield of lactic acid by different catalysts. It can be seen from Figure 5 that the peak areas of lactic acid products of all the samples of catalysts were larger than the CK sample serving as a control without any catalyst. Furthermore, as the Si/Al ratio of the β zeolite and the Sn loading increased, the components found in the chromatogram decreased significantly compared to the CK chromatogram. Notably, the compound with a retention time of 29 min had been identified as furfural, which was the primary product resulting from CK treatment. Although furfural was detected in the chromatograms of products treated with SA1 and SA2 catalysts, there was a discernible decreasing trend. With the further enhancement of the Si/Al ratio of the β zeolite and the Sn loading, the furfural peak signals became undetectable in the chromatograms of SA3, SA4, and SA5 catalysts. This is consistent with the existing research, indicating that the primary catalytic step of Sn-based catalysts involves converting furfural into lactic acid [29].

Figure 6 illustrates the glucose conversion rate and corresponding lactic acid yield for different catalysts. As shown in Figure 6a, the glucose conversion rate was 82.09% without a catalyst, whereas the lactic acid yield was merely 2.21%. This indicates that glucose is also degraded under the same hydrothermal treatment conditions during blank treatment, but the main product is furfural rather than lactic acid, so the conversion rate of lactic acid is very low. This also shows that the conversion of furfural to lactic acid is the main catalytic process for the conversion of glucose to lactic acid. When Sn-based zeolites were present, the glucose conversion rate varied from a minimum of 91.26% for the SA1 sample to a maximum of 96.59% for the SA4 sample. Figure 6b presents the lactic acid yield test results, revealing that the SA4 sample exhibited the best catalytic performance with a lactic acid yield of 41.76%, followed by the SA5 sample with a lactic acid yield of 39.42%. The catalytic efficiency of the zeolites, ranked from highest to lowest, was as follows: SA4 > SA5 > SA3 > SA2 > SA1. Based on the experimental test results, it was evident that the Sn and Al elements could synergistically catalyze the conversion of glucose into lactic acid which was consistent with the findings of Dijkmans et al. [30].

## 3. Materials and Methods

### 3.1. Materials

Tin (IV) chloride pentahydrate (SnCl_4_∙5H_2_O, ≥99%) and glucose monohydrate (AR) were purchased from Tianjin Kermel Reagent Co., (Tianjin, China). Lactate acid (LA, >98%) was purchased from TCI (Shanghai, China). Pyridine (99%) was obtained from Acros Organics, (Shanghai, China). The β zeolite was provided by Zhuoran Environmental Technology Co., (Dalian, China).

### 3.2. Catalysts Synthesis

**Figure 7:**
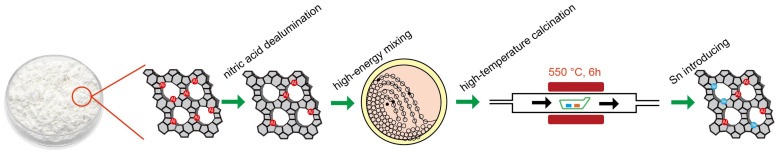
Schematic diagram of preparation of Sn-β zeolites.

The β zeolite with a weight of 5 g was mixed with different concentrations of HNO_3_, which were 0.2, 0.4, 0.6, 0.8, and 1.0 mol/L, in a mass ratio of 1:40. The mixture was added into a 250 mL round bottom flask and stirred in an oil bath at 80 °C for 6 h. After the reaction, it was cooled down and washed with deionized water until neutral, and then dried at 105 °C to remove water. The process is shown in Figure 7.

Different dealuminated β zeolite and SnCl_4_·5H_2_O were subjected to high energy mechanical ball milling for 30 min at a mass ratio of 5:1. The samples obtained were dried overnight at 50 °C, and then calcined at 550 °C at 5 °C/min for 6 h. The obtained Sn-beta zeolites were named SA1, SA2, SA3, SA4, and SA5, corresponding to the nitric acid concentrations of 0.2 mol/L, 0.4 mol/L, 0.6 mol/L, 0.8 mol/L, and 1.0 mol/L.

### 3.3. Sn-β Zeolite Characterizations

The nitrogen adsorption–desorption isotherms were measured at −196 °C, on a specific surface area tester (Autosorb-iQ, Quantochrome Instruments, Boynton Beach, FL, USA). Prior to the measurements, the Sn-β zeolite was out-gassed at 350 °C for 6 h under vacuum. From the N_2_ adsorption isotherms, the textural properties of the materials were calculated: the specific surface area (SBET) using the Brunauer–Emmett–Teller (BET) equation, and the external surface area (Sext) and micropore volume (Vmicro) using the t-plot method of the ASiQwin software (version 5.2). The Sn, Al, and Si content of zeolite were analyzed by induced coupled plasma-atomic emission spectroscopy (ICP-OES) (ICAP 700 SERIES, Agilent Technologies, Santa Clara, CA, USA). Before the ICP-OES, the Sn-β zeolite (10 mg) was digested by microwave with 1 mL of HF and 1 mL of HNO_3_ in a closed vessel at 180 °C, followed by a second digestion with HCl. The wide-angle XRD patterns (10 < 2 h < 60) were collected at room temperature by X-ray powder diffraction (D8 Advance Series, Bruker Corporation, Karlsruhe, Baden-Württemberg, Germany) with Cu Ka radiation and step size of 0.02. Fourier transform infrared (FT-IR) spectra were recorded in transmission mode as KBr pellets (where 1–2 mg sample was mixed with 100 mg KBr and then pressed into a transparent film) using an IS5 spectrophotometer (IS5, Thermos Fisher Scientific, Waltham, MA, USA). The resolution of the FTIR was set in 4 cm^−1^ and each spectrum was composed of 64 scans. Transmittance was measured with the wavenumber from 650 to 4000 cm^−1^. Before each measurement, the baseline was corrected, and the spectra were normalized. Diffuse reflectance UV–vis spectra were recorded using a solid UV detector (UV3600 Plus, Shimadzu, Kyoto, Honshu, Japan). The Fourier transform infrared (FTIR) spectroscopy measurements of the adsorbed pyridine were carried out using an FTIR spectrometer (Bruker Tenser II, Bruker Corporation, Karlsruhe, Baden-Württemberg, Germany). Self-supporting zeolite wafers were pretreated at 400 °C for 1 h under vacuum. After cooling down to room temperature (25 °C), the sample was saturated with pyridine vapor. For the pyridine FT-IR, the sample was heated to the 200 °C temperature and kept for 30 min under vacuum. The spectrum was recorded after the sample was cooled down to room temperature. Lewis acid and Brønsted acid quantities were calculated by the method of Emeis [31]. NH_3_-TPD was performed with a Thermo TPDRO Catalyst Analyzer System (Thermos Fisher Scientific, Waltham, MA, USA). The sample was activated in He flow at 300 °C for 2 h, and then equilibrated with NH_3_ gas for 1 h at room temperature and flushed with He for 1 h at 100 °C to remove physically adsorbed ammonia. The desorption of ammonia was carried out in He flows from 50 °C to 550 °C at a heating rate of 10 °C·min^−1^.

### 3.4. Catalytic Test

About 5 g of glucose was completely dissolved in 500 mL of distilled water to prepare a 1% (*w*/*v*) glucose solution as the reaction substrate.

We added glucose (10 mL) and 0.1 g of catalyst to a 20-mL Teflon-lined stainless steel autoclave reactor. After the autoclave was sealed, we heated the reactor to 190 °C at a rate of 10 °C/min and maintained this temperature for 7 h. After the reaction was complete, we used a cold-water bath to cool the reactor to ambient temperature.

The products formed in the reaction solution were filtered with a 0.22 μm filter and then identified using a high-performance liquid chromatography (HPLC; LPG-3400SD, DIONEX, Sunnyvale, CA, USA) analysis system. The conversion of glucose was analyzed with the external standard method on the HPLC equipped with an Aminex HPX-87H column (300 mm × 7.8 mm) and a refractive index detector (RI-201H, Shimadzu, Kyoto, Honshu, Japan). An aqueous H_2_SO_4_ (0.005 M) solution was used as the mobile phase, with a flow rate of 0.6 mL/min [26]. The column temperature was 55 °C. The yield of LA was analyzed using the external standard method, with UV detection (Ultimate 3000 detector, DIONEX, Sunnyvale, CA, USA) at a wavelength of 210 nm.

Glucose conversion and product yields were calculated using the following Equations (1) and (2):

(1) $$\mathrm{Glucose}\;\mathrm{conversion}=100\times \frac{\mathrm{Moles}\;\mathrm{of}\;\mathrm{glucose}\;\mathrm{reacted}}{\mathrm{Moles}\;\mathrm{of}\;\mathrm{starting}\;\mathrm{glucose}}$$

(2) $$\mathrm{LA}\;\mathrm{yield}=100\times \frac{\mathrm{Moles}\;\mathrm{of}\;C\;\mathrm{in}\;\mathrm{produced}\;\mathrm{LA}}{\mathrm{Moles}\;\mathrm{of}\;C\;\mathrm{in}\;\mathrm{glucose}}$$

All the reactions were repeated at least twice, and the difference between the two results was less than 3%.

## 4. Conclusions

The β zeolites with different Si/Al molar ratios ranging from 10 to 75 were successfully prepared by washing with nitric acid solutions of varying concentrations. The introduction of Sn into the framework of the β zeolite was achieved via physical high-energy grinding and the subsequent high-temperature calcination. The results showed that when the Si/Al ratio was 45, the Sn loading increased significantly with the increase in the Si/Al ratio. When the Si/Al ratio was above 45, the Sn load increased more gradually. By carefully controlling the degree of aluminum removal and Sn loading into the β zeolite, the number and types of acid sites could be indirectly manipulated. Overall, the removal of aluminum from the molecular sieve created vacancies in its framework, facilitating the introduction of Sn. The β zeolite with a specific Si/Al ratio was selected and hybridized with Sn to form a double solid catalyst similar to Al-β-Sn zeolite, which enhanced its catalytic efficiency. The catalytic conversion of glucose to lactic acid demonstrated that the SA4 sample with a Si/Al ratio of 45 exhibited remarkable catalytic performance. The glucose conversion rate increased from 82.09% to 92.14% while the lactic acid yield increased from 2.21% to 39.42%. The prepared Sn-β zeolites have great potential in glucose conversion and future studies can focus on the conversion process.

## Figures and Tables

**Table 1:** Si, Al, and Sn mass fraction in a variety of Sn-β zeolites.

Sample	Si (%)	Al (%)	Si/Al (Molar Ratio)	Sn (%)
**SA1**	28.35	2.56	11	0.29
**SA2**	34.73	1.81	19	0.63
**SA3**	33.51	1.02	32	1.58
**SA4**	31.95	0.68	45	1.87
**SA5**	31.81	0.42	73	1.93

**Table 2:** Physical properties of a variety of Sn-β zeolites with different Sn-β zeolites.

Sample	S_BET_ (m^2^/g)	S_micro_ (m^2^/g)	S_ext_ (m^2^/g)	V_p total_ (cm^3^/g)	V_p micro_ (cm^3^/g)	V_p mes_ (cm^3^/g)
**SA1**	628	587	41	0.57	0.30	0.27
**SA2**	664	635	29	0.44	0.32	0.12
**SA3**	684	645	39	0.66	0.33	0.33
**SA4**	680	640	40	0.66	0.32	0.34
**SA5**	476	414	62	1.05	0.65	0.41

**Table 3:** The acidity of Sn-β zeolite with different Si/Al ratios.

Sample	Lewis Acid Sites (µmol∙g^−1^) ^a^	Brønsted Acid Sites (µmol∙g^−1^) ^a^	Acidic Site Distribution Based on NH_3_-TPD (mmol∙g^−1^) ^b^	
			Weak and Medium 50–370 °C	Strong 370–550 °C
**SA1**	82.417	21.884	0.366	0.187
**SA2**	86.932	22.465	0.841	0.279
**SA3**	163.540	35.481	1.003	0.316
**SA4**	234.442	79.837	1.008	0.340
**SA5**	103.325	20.460	0.807	0.290

^a^: Lewis acid and Brønsted acid quantities were calculated from pyridine FT-IR. ^b^: weak–medium and strong acidic sites were obtained based on NH_3_-TPD.

## Data Availability

The data presented in this study are available from the corresponding author upon request.

## References

[1] de Clippel F., Dusselier M., Van Rompaey R., Vanelderen P., Dijkmans J., Makshina E., Giebeler L., Oswald S., Baron G.V., Denayer J.F.M. (2012). Fast and Selective Sugar Conversion to Alkyl Lactate and Lactic Acid with Bifunctional Carbon–Silica Catalysts. J. Am. Chem. Soc..

[2] Ajala E.O., Olonade Y.O., Ajala M.A., Akinpelu G.S. (2020). Lactic Acid Production from Lignocellulose—A Review of Major Challenges and Selected Solutions. ChemBioEng Rev..

[3] Perez-Pariente J., Martens J.A., Jacobs P.A. (1987). Crystallization Mechanism of Zeolite Beta from (TEA)2O, Na_2_O and K_2_O Containing Aluminosilicate Gels. Appl. Catal..

[4] Möller K., Bein T. (2011). Pores Within Pores—How to Craft Ordered Hierarchical Zeolites. Science.

[5] Katkar S.S., Mohite P.H., Gadekar L.S., Vidhate K.N., Lande M.K. (2010). ZnO-Beta Zeolite: As an Effective and Eco-Friendly Heterogeneous Catalyst for the Synthesis of Benzothiazole Derivatives. Chin. Chem. Lett..

[6] Li Y., Luo C., Wang X., Huang C., Chen B. (2014). Transalkylation of Multi-Secbutylbenzenes with Benzene over Hierarchical Beta Zeolite. Chin. J. Chem. Eng..

[7] Elliot S.G., Andersen C., Tolborg S., Meier S., Sádaba I., Daugaard A.E., Taarning E. (2017). Synthesis of a Novel Polyester Building Block from Pentoses by Tin-Containing Silicates. RSC Adv..

[8] Meng Q., Liu J., Xiong G., Liu X., Liu L., Guo H. (2018). Aerosol-Seed-Assisted Hydrothermal Synthesis of Sn-Beta Zeolite and Its Catalytic Performance in Baeyer–Villiger Oxidation. Microporous Mesoporous Mater..

[9] Holm M.S., Pagán-Torres Y.J., Saravanamurugan S., Riisager A., Dumesic J.A., Taarning E. (2012). Sn-Beta Catalysed Conversion of Hemicellulosic Sugars. Green Chem..

[10] Pescarmona P.P., Janssen K.P.F., Delaet C., Stroobants C., Houthoofd K., Philippaerts A., De Jonghe C., Paul J.S., Jacobs P.A., Sels B.F. (2010). Zeolite-Catalysed Conversion of C3 Sugars to Alkyl Lactates. Green Chem..

[11] Hammond C., Conrad S., Hermans I. (2012). Simple and Scalable Preparation of Highly Active Lewis Acidic Sn-β. Angew. Chem. Int. Ed..

[12] Serrano D.P., Van Grieken R., Sánchez P., Sanz R., Rodríguez L. (2001). Crystallization Mechanism of All-Silica Zeolite Beta in Fluoride Medium. Microporous Mesoporous Mater..

[13] Corma A., Nemeth L.T., Renz M., Valencia S. (2001). Sn-Zeolite Beta as a Heterogeneous Chemoselective Catalyst for Baeyer–Villiger Oxidations. Nature.

[14] Yang X., Wang L., Lu T., Gao B., Su Y., Zhou L. (2020). Seed-Assisted Hydrothermal Synthesis of Sn-Beta for Conversion of Glucose to Methyl Lactate: Effects of the H2O Amount in the Gel and Crystallization Time. Catal. Sci. Technol..

[15] Yang X., Liu Y., Li X., Ren J., Zhou L., Lu T., Su Y. (2018). Synthesis of Sn-Containing Nanosized Beta Zeolite As Efficient Catalyst for Transformation of Glucose to Methyl Lactate. ACS Sustain. Chem. Eng..

[16] Camblor M.A., Villaescusa L.A., Díaz-Cabañas M.J. (1999). Synthesis of All-Silica and High-Silica Molecular Sieves in Fluoride Media. Top. Catal..

[17] Taarning E., Saravanamurugan S., Spangsberg Holm M., Xiong J., West R.M., Christensen C.H. (2009). Zeolite-Catalyzed Isomerization of Triose Sugars. ChemSusChem.

[18] Dong W., Shen Z., Peng B., Gu M., Zhou X., Xiang B., Zhang Y. (2016). Selective Chemical Conversion of Sugars in Aqueous Solutions without Alkali to Lactic Acid Over a Zn-Sn-Beta Lewis Acid-Base Catalyst. Sci. Rep..

[19] Xia M., Dong W., Shen Z., Xiao S., Chen W., Gu M., Zhang Y. (2020). Efficient Production of Lactic Acid from Biomass-Derived Carbohydrates under Synergistic Effects of Indium and Tin in In–Sn-Beta Zeolites. Sustain. Energy Fuels.

[20] Shen Z., Kong L., Zhang W., Gu M., Xia M., Zhou X., Zhang Y. (2019). Surface Amino-Functionalization of Sn-Beta Zeolite Catalyst for Lactic Acid Production from Glucose. RSC Adv..

[21] Holm M.S., Saravanamurugan S., Taarning E. (2010). Conversion of Sugars to Lactic Acid Derivatives Using Heterogeneous Zeotype Catalysts. Science.

[22] Antunes M.M., Lima S., Neves P., Magalhães A.L., Fazio E., Fernandes A., Neri F., Silva C.M., Rocha S.M., Ribeiro M.F. (2015). One-Pot Conversion of Furfural to Useful Bio-Products in the Presence of a Sn,Al-Containing Zeolite Beta Catalyst Prepared via Post-Synthesis Routes. J. Catal..

[23] Vega-Vila J.C., Harris J.W., Gounder R. (2016). Controlled Insertion of Tin Atoms into Zeolite Framework Vacancies and Consequences for Glucose Isomerization Catalysis. J. Catal..

[24] Kiricsi I., Flego C., Pazzuconi G., Parker W.O., Millini R., Perego C., Bellussi G. (1994). Progress toward Understanding Zeolite.Beta. Acidity: An IR and 27Al NMR Spectroscopic Study. J. Phys. Chem..

[25] Li P., Liu G., Wu H., Liu Y., Jiang J., Wu P. (2011). Postsynthesis and Selective Oxidation Properties of Nanosized Sn-Beta Zeolite. J. Phys. Chem. C.

[26] Lu T., Fu X., Zhou L., Su Y., Yang X., Han L., Wang J., Song C. (2017). Promotion Effect of Sn on Au/Sn-USY Catalysts for One-Pot Conversion of Glycerol to Methyl Lactate. ACS Catal..

[27] Shen Z., Chen W., Zhang W., Gu M., Dong W., Xia M., Si H., Zhang Y. (2022). Efficient Catalytic Conversion of Glucose into Lactic Acid over Y-β and Yb-β Zeolites. ACS Omega.

[28] Zhou L., Shi M., Cai Q., Wu L., Hu X., Yang X., Chen C., Xu J. (2013). Hydrolysis of Hemicellulose Catalyzed by Hierarchical H-USY Zeolites—The Role of Acidity and Pore Structure. Microporous Mesoporous Mater..

[29] Bing L., Zhang Z., Deng K. (2012). Efficient One-Pot Synthesis of 5-(Ethoxymethyl)Furfural from Fructose Catalyzed by a Novel Solid Catalyst. Ind. Eng. Chem. Res..

[30] Dijkmans J., Dusselier M., Gabriëls D., Houthoofd K., Magusin P.C.M.M., Huang S., Pontikes Y., Trekels M., Vantomme A., Giebeler L. (2015). Cooperative Catalysis for Multistep Biomass Conversion with Sn/Al Beta Zeolite. ACS Catal..

[31] Emeis C.A. (1993). Determination of Integrated Molar Extinction Coefficients for Infrared Absorption Bands of Pyridine Adsorbed on Solid Acid Catalysts. J. Catal..

